# Characterising smoking and nicotine use behaviours among women of reproductive age: a 10-year population study in England

**DOI:** 10.1186/s12916-024-03311-4

**Published:** 2024-04-18

**Authors:** Sarah E. Jackson, Jamie Brown, Caitlin Notley, Lion Shahab, Sharon Cox

**Affiliations:** 1 Department of Behavioural Science and Health, University College London, London, UK; 2 SPECTRUM Consortium, Edinburgh, UK; 3 Faculty of Medicine and Health Sciences, Norwich Medical School, Lifespan Health Research Centre, University of East Anglia, Norwich, UK

**Keywords:** Women, Female, Reproductive age, Childbearing age, Smoking, Nicotine use

## Abstract

**Background:**

Tobacco smoking affects women’s fertility and is associated with substantial risks of adverse pregnancy outcomes. This study explored trends by socioeconomic position in patterns of smoking, use of non-combustible nicotine products, and quitting activity among women of reproductive age in England.

**Methods:**

Data come from a nationally representative monthly cross-sectional survey. Between October 2013 and October 2023, 197,266 adults (≥ 18 years) were surveyed, of whom 44,052 were women of reproductive age (18–45 years). Main outcome measures were current smoking, vaping, and use of nicotine replacement therapy (NRT), heated tobacco products (HTPs), and nicotine pouches; mainly/exclusively smoking hand-rolled cigarettes and level of dependence among current smokers; past-year quit attempts among past-year smokers; and success of quit attempts among those who tried to quit. We modelled time trends in these outcomes, overall and by occupational social grade (ABC1 = more advantaged/C2DE = less advantaged).

**Results:**

Smoking prevalence among women of reproductive age fell from 28.7% [95%CI = 26.3–31.2%] to 22.4% [19.6–25.5%] in social grades C2DE but there was an uncertain increase from 11.7% [10.2–13.5%] to 14.9% [13.4–16.6%] in ABC1. By contrast, among all adults and among men of the same age, smoking prevalence remained relatively stable in ABC1. Vaping prevalence among women of reproductive age more than tripled, from 5.1% [4.3–6.0%] to 19.7% [18.0–21.5%], with the absolute increase more pronounced among those in social grades C2DE (reaching 26.7%; 23.3–30.3%); these changes were larger than those observed among all adults but similar to those among men of the same age. The proportion of smokers mainly/exclusively smoking hand-rolled cigarettes increased from 40.5% [36.3–44.9%] to 61.4% [56.5–66.1%] among women of reproductive age; smaller increases were observed among all adults and among men of the same age. Patterns on other outcomes were largely similar between groups.

**Conclusions:**

Among women of reproductive age, there appears to have been a rise in smoking prevalence in the more advantaged social grades over the past decade. Across social grades, there have been substantial increases in the proportion of women of reproductive age who vape and shifts from use of manufactured to hand-rolled cigarettes among those who smoke. These changes have been more pronounced than those observed in the general adult population over the same period.

**Supplementary Information:**

The online version contains supplementary material available at 10.1186/s12916-024-03311-4.

## Background

Tobacco smoking is the single largest cause of premature mortality and morbidity and for some groups carries extra risks. For women of reproductive age (15–45 years [1]) risks include reduced fertility, and for women who are pregnant smoking increases the chances of complications, miscarriage, and premature birth, and post-partum is associated with adverse infant health outcomes [2–4]. Children whose parents smoke face greater exposure to the effects of second-hand smoke and are more likely to take up smoking themselves [5, 6]. Reducing smoking in pregnancy has been identified as a priority for tobacco control activity [7, 8] and has attracted considerable research attention [3]. However, much of the harm associated with smoking in pregnancy could be prevented by reducing smoking among women of reproductive age before they become pregnant. There is good evidence from representative population surveys on the prevalence and patterns of smoking in the adult population in England [9]. However, less is known about women of reproductive age specifically. Understanding patterns of smoking, levels of dependence, and quitting activity in this target group and how they are changing over time can inform the development of interventions and targeting of resources.

In addition to the substantial, well-established risks of smoking during pregnancy, there are also likely (albeit lower) risks associated with use of non-combustible nicotine products [10]. A range of non-combustible nicotine products are available in England — including nicotine replacement therapy (NRT), nicotine vaping products (often referred to as e-cigarettes or vapes), heated tobacco products (HTPs), and nicotine pouches — which deliver nicotine without most of the harmful components of tobacco smoke. Evidence suggests that using non-combustible nicotine products during pregnancy poses considerably lower risks for adverse outcomes than smoking, with NRT likely providing the greatest reduction, but that any use of nicotine is likely to be worse for the developing foetus than none [10–15]. It is therefore important to monitor use of non-combustible nicotine products among women of reproductive age.

In examining smoking and non-combustible nicotine use among women of reproductive age, it is important to consider differences across socioeconomic groups. Smoking is a socioeconomically patterned behaviour: people from less advantaged groups are much more likely to smoke, show greater signs of dependence, and experience disproportionate levels of harm from smoking [16]. This disparity is particularly pronounced for smoking in pregnancy. Compared with women from advantaged backgrounds, those from disadvantaged backgrounds are not only more likely to smoke before pregnancy, but are also less likely to quit in pregnancy, and among those who quit, more likely to resume smoking after birth [17, 18].

This study aimed to characterise patterns of smoking, cigarette dependence, quitting activity, and use of non-combustible nicotine over the past decade among women of reproductive age in England, and obtain up-to-date estimates of these in 2023. A secondary aim was to explore differences by socioeconomic position. Specific research questions (RQs) were:

1. Among women of reproductive age in England, to what extent have there been changes between 2013 and 2023 in:
  - The prevalence of smoking, nicotine vaping, and use of NRT, HTPs, and nicotine pouches;
  - The main type of cigarettes smoked (manufactured/hand-rolled) and levels of cigarette dependence, among those who currently smoke;
  - Rates of quit attempts, among those who have smoked regularly in the past year; and
  - Success in quitting, among those who have made an attempt to stop smoking in the past year?
2. To what extent have these changes differed by socioeconomic position (indexed by occupational social grade)?
3. How far do results for RQ1-3 reflect what has occurred across the entire adult population in England over this period?

## Methods

### Pre-registration

The study protocol and analysis plan were pre-registered on Open Science Framework (https://osf.io/em8g2). We made one amendment prior to peer review. We had planned to analyse time trends with survey month modelled using restricted cubic splines with five knots. However, for analyses of trends in current use of nicotine pouches, we reduced this to three knots to avoid overfitting, because pouch use was only assessed over a relatively short period (November 2020–October 2023) and prevalence was assumed to be zero before this, based on previous evidence [19].

### Design

Data were drawn from the Smoking Toolkit Study, an ongoing monthly cross-sectional survey of a nationally representative representative sample of adults (≥ 16 years) in England [20]. The study uses a hybrid of random probability and simple quota sampling to select a new sample of approximately 1700 adults each month. Interviews are held with one household member in selected geographic output areas until quotas are fulfilled. The quotas are based on factors influencing the probability of being at home (i.e. working status, age and gender). This hybrid form of random probability and quota sampling is considered superior to conventional quota sampling. Here, the choice of households to approach is limited by the random allocation of small output areas and rather than being sent to specific households in advance, interviewers can choose which households within these small geographic areas are most likely to fulfil their quotas. Therefore, unlike random probability sampling, it is not appropriate to record the response rate in the Smoking Toolkit Study.

Data were collected monthly through face-to-face computer-assisted interviews up to February 2020. However, social distancing restrictions under the COVID-19 pandemic meant that no data were collected in March 2020, and data from April 2020 onwards have been collected via telephone. The telephone-based data collection relies upon the same combination of random location and quota sampling, and weighting approach as the face-to-face interviews and comparisons of the two data collection modalities indicate good comparability [21–23].

For the present study, we used data from respondents to the monthly survey over a 10-year period from October 2013 to October 2023 (the most recent data available at the time of analysis). We restricted the sample to those aged ≥ 18 years as 16 and 17-year-olds were not surveyed in all waves. Our primary focus was women of reproductive age (which we defined as per the Office for National Statistics [1] as up to 45 years). We also provided data on these outcomes among the entire adult population in England for context.

### Measures

#### Smoking status

Participants were asked which of the following best applies to them:

- I smoke cigarettes (including hand-rolled) every day
- I smoke cigarettes (including hand-rolled), but not every day
- I do not smoke cigarettes at all, but I do smoke tobacco of some kind (e.g. pipe, cigar or shisha)
- I have stopped smoking completely in the last year
- I stopped smoking completely more than a year ago
- I have never been a smoker (i.e. smoked for a year or more)

For analyses of current smoking, those who responded *a-c* were considered current smokers (coded 1) and those who responded *d*-*f* non-smokers (coded 0). For (unplanned) analyses of non-daily smoking (see statistical analysis section), those who responded *b* were considered non-daily smokers (coded 1) and all others (i.e. daily smokers or non-smokers) were coded 0. For analyses of quit attempts, those who responded *a-d* were considered past-year smokers and those who responded *e-f* were excluded.

#### Use of non-combustible nicotine products

Several questions asked participants about use of a range of nicotine products. Current smokers were asked ‘Do you regularly use any of the following in situations when you are not allowed to smoke?’; past-year smokers were asked ‘Can I check, are you using any of the following either to help you stop smoking, to help you cut down or for any other reason at all?’; and non-smokers were asked ‘Can I check, are you using any of the following?’. Those who reported using e-cigarettes in response to any of these questions were considered current vapers; those who reported using NRT (nicotine gum, lozenges/tablets, inhaler, nasal spray, patch, or mouth spray) current NRT users; those who reported using HTPs (‘heat-not-burn cigarette (e.g. iQOS, heatsticks)’) current HTP users; and those who reported using nicotine pouches (‘tobacco-free nicotine pouch/pod or ‘white pouches’ that you place on your gum’) current nicotine pouch users.

HTPs were included in the list of response options from December 2016 and nicotine pouches from November 2020; given the low prevalence of use of these products [19, 24], we imputed missing values as 0 (indicating no use) for participants surveyed before the response options were introduced. As a sensitivity check, we reran these models from the time when these data were available (i.e. December 2016 onwards for HTPs and November 2020 onwards for pouches); the results were unchanged.

#### Main type of cigarettes smoked

Current smokers were asked ‘How many cigarettes per day do you usually smoke?’ and ‘How many of these do you think are hand-rolled?’. Main type of cigarettes smoked was defined as hand-rolled for those reporting at least 50% of their total cigarette consumption is hand-rolled, and manufactured for those reporting that less than 50% is hand-rolled. This definition has been used in previous studies [25–27] and allows inclusion of those who smoke both hand-rolled and manufactured cigarettes.

#### Level of cigarette dependence

Current smokers were asked to self-report ratings of the strength of urges to smoke over the past 24 h [not at all (coded 0), slight (1), moderate (2), strong (3), very strong (4) and extremely strong (5)]. This variable was also coded ‘0’ for smokers who responded ‘not at all’ to the (separate) question: ‘How much of the time have you spent with the urge to smoke?’ [28]. This measure has been validated and performs at least as well as the Fagerström Test of Cigarette Dependence and the Heaviness of Smoking Index in predicting smoking cessation while not being subject to bias due to population-level changes in cigarette consumption over the time period of the study [28]. Scores were skewed towards lower values so we log-transformed this variable for analysis (with values of 0 imputed as 0.01 before the transformation was applied) and reported results as geometric means.

#### Quit attempts

Past-year smokers were asked: ‘How many serious attempts to stop smoking have you made in the last 12 months? By serious attempt I mean you decided that you would try to make sure you never smoked again. Please include any attempt that you are currently making and please include any successful attempt made within the last year’. Those who reported making at least one serious quit attempt in the past year were coded 1, else they were coded 0.

#### Success of quit attempts

Past-year smokers who had made an attempt to quit in the past year were asked: ‘How long did your most recent serious quit attempt last before you went back to smoking?’ Those who reported that they were still not smoking were coded 1, else they were coded 0.

#### Occupational social grade

Occupational social grade was defined according to the National Readership Survey classification [29] and categorised as ABC1 (includes managerial, professional, and upper supervisory occupations) and C2DE (includes manual routine, semi-routine, lower supervisory, and long-term unemployed). This occupational measure of social grade is a valid index of SES, widely used in research in UK populations, which is particularly relevant in the context of tobacco use [30].

### Statistical analysis

Data were analysed in R version 4.2.1. Participants with missing data on key variables were excluded on a per-analysis basis (see Table 1 footnote for details). The Smoking Toolkit Study uses raking to weight the sample to match the population of England in terms of key demographics. These key demographics are determined each month using data from the UK Census, the Office for National Statistics mid-year estimates, and the National Readership Survey [20]. The following analyses used weighted data.

**Table 1:** Modelled estimates of changes in smoking, use of non-combustible nicotine products, smoking characteristics, and quitting activity among women of reproductive age compared with all adults in England, from October 2013 to October 2023

	**Overall**			**Social grades ABC1 (more advantaged)**			**Social grades C2DE (less advantaged)**		
	***N*** ^**a**^	**October 2013** ^**b**^	**October 2023** ^**b**^	***N*** ^**a**^	**October 2013** ^**b**^	**October 2023** ^**b**^	***N*** ^**a**^	**October 2013** ^**b**^	**October 2023** ^**b**^
**Current smoking**, % [95%CI]									
Women of reproductive age	43,911	19.9 [18.5–21.5]	18.0 [16.5–19.6]	26,202	11.7 [10.2–13.5]	14.9 [13.4–16.6]	17,709	28.7 [26.3–31.2]	22.4 [19.6–25.5]
All adults	196,678	19.2 [18.5–20.0]	17.0 [16.3–17.7]	118,439	12.7 [11.9–13.6]	13.2 [12.4–13.9]	78,239	27.2 [26.0–28.4]	21.9 [20.6–23.3]
**Current vaping**, % [95%CI]									
Women of reproductive age	44,052	5.1 [4.3–6.0]	19.7 [18.0–21.5]	26,287	3.6 [2.7–4.7]	14.9 [13.3–16.8]	17,765	6.7 [5.5–8.2]	26.7 [23.3–30.3]
All adults	197,266	4.5 [4.2–4.9]	13.2 [12.5–14.0]	118,799	3.6 [3.1–4.0]	10.4 [9.7–11.2]	78,467	5.7 [5.1–6.4]	16.9 [15.6–18.2]
**Current NRT use**, % [95%CI]									
Women of reproductive age	44,052	2.9 [2.3–3.6]	2.5 [2.0–3.3]	26,287	2.2 [1.5–3.2]	2.1 [1.6–2.9]	17,765	3.6 [2.8–4.8]	3.1 [2.1–4.7]
All adults	197,266	3.2 [2.9–3.5]	2.9 [2.6–3.2]	118,799	2.4 [2.0–2.9]	2.4 [2.1–2.8]	78,467	4.1 [3.6–4.8]	3.5 [2.9–4.1]
**Current HTP use** ^**c**^, % [95%CI]									
Women of reproductive age	44,052	0.0 [0.0–0.0]	0.5 [0.2–1.0]	-	-	-	-	-	-
All adults	197,266	0.0 [0.0–0.0]	0.2 [0.1–0.3]	-	-	-	-	-	-
**Current pouch use** ^**c**^, % [95%CI]									
Women of reproductive age	44,052	0.0 [0.0–0.0]	0.7 [0.4–1.2]	-	-	-	-	-	-
All adults	197,266	0.0 [0.0–0.0]	0.4 [0.3–0.6]	-	-	-	-	-	-
**Mainly smokes hand-rolled cigarettes** ^d^, % [95%CI]									
Women of reproductive age	7967	40.5 [36.3–44.9]	61.4 [56.5–66.1]	3350	35.3 [28.5–42.8]	52.5 [46.0–59.0]	4617	42.8 [37.5–48.3]	68.4 [61.5–74.5]
All adults	30,133	41.8 [39.5–44.1]	54.4 [52.0–56.9]	13,180	37.2 [33.6–41.0]	49.6 [46.3–52.9]	16,953	44.2 [41.4–47.1]	58.1 [54.6–61.5]
**Level of dependence** ^e^, geometric mean [95%CI]									
Women of reproductive age	8640	1.26 [1.10–1.45]	0.88 [0.74–1.04]	3710	1.25 [0.98–1.61]	0.63 [0.49–0.80]	4930	1.25 [1.05–1.48]	1.21 [0.96–1.52]
All adults	32,987	1.16 [1.07–1.25]	0.88 [0.80–0.96]	14,748	1.08 [0.95–1.23]	0.76 [0.67–0.86]	18,239	1.20 [1.09–1.32]	0.99 [0.87–1.13]
**Quit attempts** ^f^, % [95%CI]									
Women of reproductive age	9412	44.2 [40.0–48.3]	44.9 [40.6–49.3]	4160	45.3 [38.4–52.4]	39.1 [33.9–44.7]	5252	43.8 [38.7–49.0]	49.7 [43.1–56.3]
All adults	35,456	37.5 [35.5–39.7]	36.6 [34.5–38.8]	16,225	41.1 [37.7–44.6]	34.8 [32.1–37.6]	19,231	35.3 [32.7–38.0]	38.0 [34.8–41.2]
**Quit success** ^g^, % [95%CI]									
Women of reproductive age	3629	18.8 [14.1–24.5]	31.8 [25.6–38.7]	1649	21.1 [14.1–30.4]	28.2 [21.0–36.7]	1980	17.6 [11.9–25.3]	35.1 [25.8–45.8]
All adults	11,923	16.8 [14.3–19.7]	25.4 [22.2–28.9]	5736	18.0 [14.3–22.5]	24.6 [20.6–29.1]	6187	16.0 [12.8–19.9]	26.2 [21.5–31.5]

*Unmodelled estimates within each survey year are provided in *Additional file 1*: Table S1*

*CI* confidence interval, *HTP* heated tobacco products, *NRT* nicotine replacement therapy

^a^Unweighted sample size for each analysis. Note that there were some missing data on certain variables (smoking status *n* = 141; main type of cigarettes smoked *n* = 460; level of dependence *n* = 118; quit attempts *n* = 324); sample sizes show the number of participants contributing data to each analysis

^b^Data for October 2013 and October 2023 are weighted estimates of prevalence in these months (the first and last in the study period) from logistic regression with survey month modelled non-linearly using restricted cubic splines (five knots; three knots for analyses of current pouch use)

^c^Trends in use of HTPs and nicotine pouches were not modelled by occupational social grade, because total numbers of women of reproductive age using these products across the study period were very small (HTPs: *n* = 39 ABC1, *n* = 33 C2DE; pouches: *n* = 35 ABC1, *n* = 8 C2DE)

^d^Among current cigarette smokers

^e^Among current smokers

^f^Among past-year smokers

^g^Among past-year smokers who made a past-year quit attempt

Where there were sufficient data, we used regression models (logistic/linear as appropriate, using the ‘svyglm’ command) to estimate monthly time trends in each outcome among women of reproductive age, overall and by occupational social grade. For the overall analysis, models only included time as an independent variable. For the analysis by occupational social grade, models included time, social grade, and their interaction as independent variables — thus allowing for time trends to differ across social grades. Time (survey wave) was coded 1…n where n was the total number of months in the time series (including March 2020 when no data were collected). Time was modelled continuously using restricted cubic splines with five knots (placed at equal quantiles of the data), to allow relationships with time to be flexible and non-linear, while avoiding categorisation. We were unable to model the interaction between time and occupational social grade for use of HTPs and nicotine pouches because very few women of reproductive age in the sample reported using these products at this time. We repeated these models using data from all adults (≥ 18 years) in England, to provide context.

We used predicted estimates from our models to (i) plot the prevalence (or geometric mean, for level of cigarette dependence) of each outcome over the study period (overall and by social grade, among women of reproductive age and in the entire adult population), and (ii) derive up-to-date estimates of the prevalence of each outcome in October 2023. We followed the ‘New Statistics’ approach to reporting and interpretation of results [31, 32], focusing on effect sizes and confidence intervals rather than dichotomous thinking about statistical significance (i.e. whether a result is significant or not significant, based on an arbitrary threshold). Where confidence intervals overlap, we report changes as ‘uncertain’.

In addition to our pre-registered analyses, where there was evidence that the trend in an outcome among women of reproductive age differed from the trend in the entire adult population, we repeated the model among men of the same age (18–45 years). This allowed us to explore whether the difference in trends was due to age more generally or was specific to women of reproductive age. We also added two unplanned analyses following peer review. In the first, we modelled time trends in non-daily smoking, to explore whether changes in current smoking we observed may have been driven by changes in non-daily smoking specifically. In the second, we modelled time trends in dual use of tobacco and non-combustible nicotine (i.e. current smoking and current use of e-cigarettes, NRT, HTPs, or nicotine pouches) as an additional outcome.

## Results

A total of 197,266 (unweighted) adults aged ≥ 18 years were surveyed between October 2013 and October 2023 (weighted mean age = 47.9 years, 50.8% women, 44.6% social grades C2DE). Of these, 44,052 (unweighted) were women of reproductive age (18–45 years; weighted mean age = 31.5 years, 44.7% social grades C2DE).

### Trends in smoking and use of non-combustible nicotine products

Table 1 summarises modelled changes in smoking and use of non-combustible nicotine products between October 2013 and October 2023 among women of reproductive age compared with all adults in England.

**Fig. 1:**
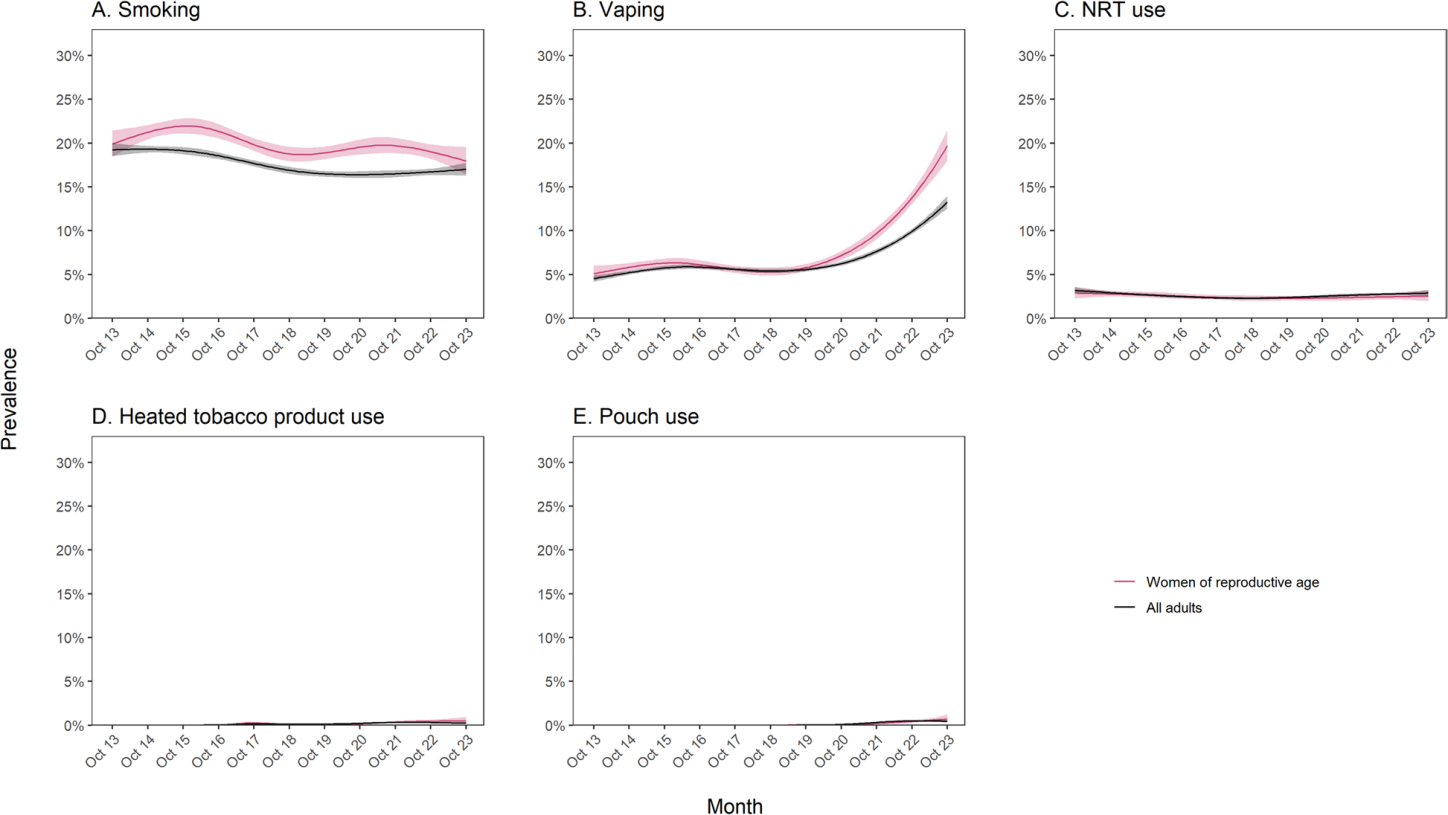
Trends in smoking and use of non-combustible nicotine products among women of reproductive age compared with all adults in England, October 2013 to October 2023. Panels show trends in the prevalence of (**A**) smoking, (**B**) vaping, and use of (**C**) nicotine replacement therapy (NRT), (**D**) heated tobacco products, and (**E**) nicotine pouches. Lines represent modelled weighted prevalence by monthly survey wave, modelled non-linearly using restricted cubic splines (five knots; three knots for nicotine pouch use). Shaded bands represent 95% confidence intervals. Corresponding figures showing trends in smoking, vaping, and NRT use stratified by occupational social grade are provided in Fig. 2

**Fig. 2:**
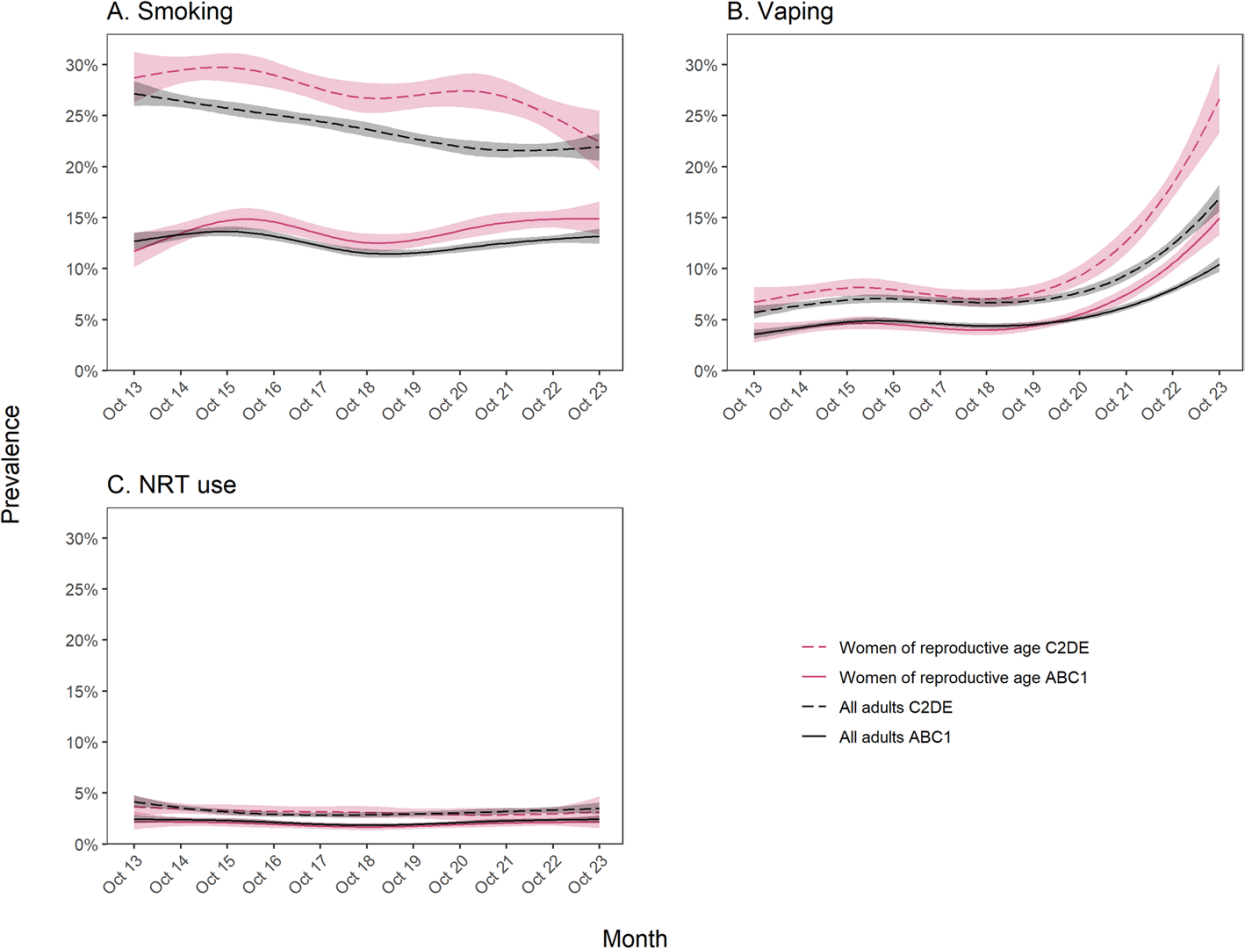
Trends in smoking and use of non-combustible nicotine products among women of reproductive age compared with all adults in England, October 2013 to October 2023 — stratified by occupational social grade. Panels show trends in the prevalence of (**A**) smoking, (**B**) vaping, and (**C**) use of nicotine replacement therapy (NRT), stratified by occupational social grade. ABC1 = more advantaged, C2DE = less advantaged. Lines represent modelled weighted prevalence by monthly survey wave, modelled non-linearly using restricted cubic splines (five knots). Shaded bands represent 95% confidence intervals

There was an uncertain decline in smoking prevalence among women of reproductive age, from 19.9% [95%CI 18.5–21.5%] to 18.0% [16.5–19.6%] (Fig. 1A). A similar decline was observed among all adults, from 19.2% [18.5–20.0%] to 17.0% [16.3–17.7%]. In both women of reproductive age and all adults, smoking prevalence was consistently higher among those from less advantaged social grades (C2DE; Fig. 2A). However, changes over time differed, with an uncertain rise in smoking prevalence from 11.7% [10.2–13.5%] to 14.9% [13.4–16.6%] among women of reproductive age from more advantaged social grades (ABC1) and a considerable decline from 28.7% [26.3–31.2%] to 22.4% [19.6–25.5%] among those from less advantaged social grades (Fig. 2A). By contrast, among all adults (Fig. 2A) and among men of the same age (Additional file 1: Fig. S1), smoking prevalence remained relatively stable among those from more advantaged social grades. Unplanned analyses indicated this pattern of results was not driven by non-daily smoking specifically: trends in non-daily smoking were similar between women of reproductive age and all adults and across social grades (Additional file 1: Table S2; Additional file 1: Fig. S2).

The prevalence of vaping more than tripled among women of reproductive age, from 5.1% [4.3–6.0%] to 19.7% [18.0–21.5%] (Fig. 1B). A similar increase in vaping was observed among men of the same age, from 5.8% [5.0–6.8%] to 20.2% [18.5–22.1%] (Additional file 1: Fig. S3A) and a smaller, but substantial, increase among all adults, from 4.5% [4.2–4.9%] to 13.2% [12.5–14.0%] (Fig. 1B). These increases predominantly occurred between 2020 and 2023. As a result, in October 2023 vaping prevalence was higher among adults aged 18-45 compared with the entire adult population. In women of reproductive age, men of the same age, and the adult population in general, vaping prevalence was consistently higher among those from less advantaged social grades, and the absolute rise in prevalence was larger — reaching 26.7% in October 2023 among women of reproductive age from less advantaged social grades compared with 10.4% among those from more advantaged social grades (Fig. 2A, Additional file 1: Fig. S3B).

The prevalence of NRT use among women of reproductive age was similar to prevalence among all adults, and was relatively stable over time at approximately 3% (Fig. 1C). NRT use was slightly more prevalent among those from less vs. more advantaged social grades and did not change substantially over time in either group (Fig. 2C).

Use of HTPs and nicotine pouches increased by a small amount, both among women of reproductive age and among all adults, but remained rare (< 1%) across the study period (Fig. 1D and E). There were insufficient numbers using these products to model time trends by social grade.

Trends in the prevalence of dual use followed a similar pattern to trends in vaping prevalence. The proportion of women of reproductive age using both tobacco and non-combustible nicotine increased from 6.2% [5.3–7.2%] to 9.5% [8.3–10.9%] across the study period (Additional file 1: Table S2; Additional file 1: Fig. S4). There was a similar rise among men of the same age (Additional file 1: Fig. S5) and a smaller but significant rise among all adults (Additional file 1: Fig. S4).

### Trends in smoking characteristics and quitting activity

Table 1 summarises modelled changes in smoking characteristics and quitting activity between October 2013 and October 2023.

**Fig. 3:**
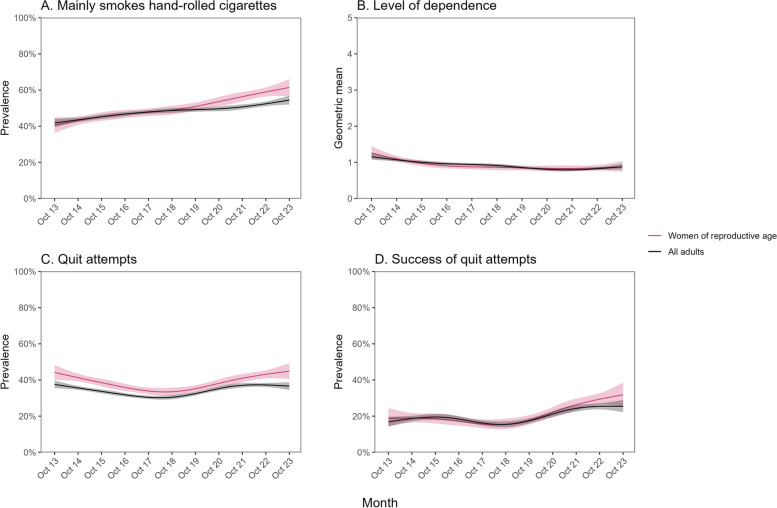
Trends in smoking characteristics and quitting activity among women of reproductive age compared with all adults in England, October 2013 to October 2023, Panels show trends in (**A**) the proportion of current cigarette smokers mainly smoking hand-rolled (vs. manufactured) cigarettes, (**B**) the geometric mean level of dependence among current smokers, (**C**) the proportion of past-year smokers making ≥ 1 past-year quit attempt, and (**D**) the proportion of past-year smokers who made ≥ 1 past-year quit attempt who were still not smoking at the time of the survey. Lines represent modelled weighted prevalence (or mean, for level of dependence) by monthly survey wave, modelled non-linearly using restricted cubic splines (five knots). Shaded bands represent 95% confidence intervals. Corresponding figures showing trends stratified by occupational social grade are provided in Fig. 4

**Fig. 4:**
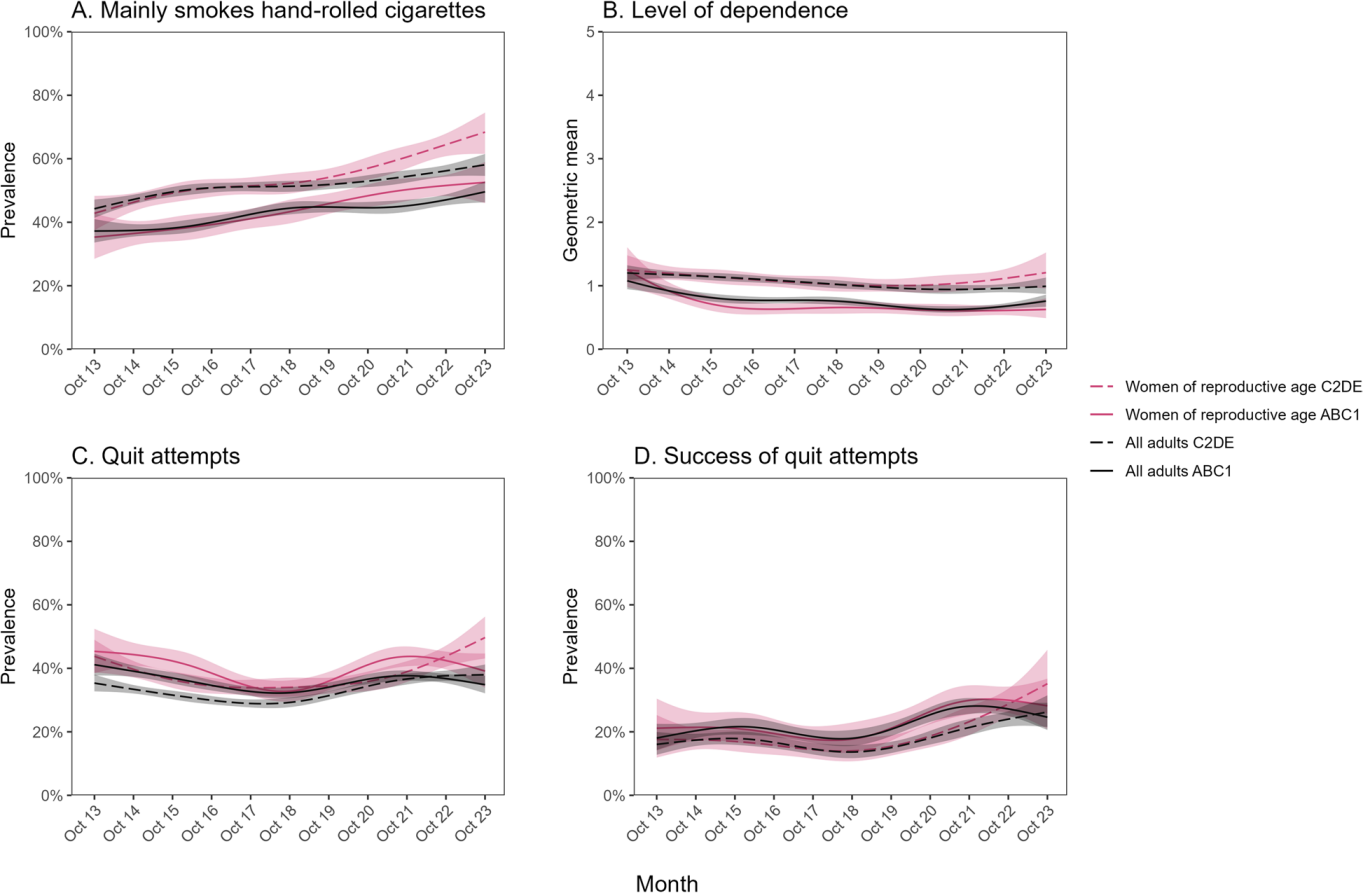
Trends in smoking characteristics and quitting activity among women of reproductive age compared with all adults in England, October 2013 to October 2023 — by occupational social grade. Panels show trends in (**A**) the proportion of current cigarette smokers mainly smoking hand-rolled (vs. manufactured) cigarettes, (**B**) the geometric mean level of dependence among current smokers, (**C**) the proportion of past-year smokers making ≥ 1 past-year quit attempt, and (**D**) the proportion of past-year smokers who made ≥ 1 past-year quit attempt who were still not smoking at the time of the survey, stratified by occupational social grade. ABC1 = more advantaged, C2DE = less advantaged. Lines represent modelled weighted prevalence (or mean, for level of dependence) by monthly survey wave, modelled non-linearly using restricted cubic splines (five knots). Shaded bands represent 95% confidence intervals

The proportion of current cigarette smokers who reported mainly or exclusively smoking hand-rolled cigarettes increased from 40.5% [36.3–44.9%] to 61.4% [56.5–66.1%] among women of reproductive age (Fig. 3A). This increase was slightly larger than the increase observed among all adults, from 41.8% [39.5–44.1%] to 54.4% [52.0–56.9%] (Fig. 3A), and among men of the same age, from 49.2% [45.1–53.4%] to 62.3% [57.6–66.7%] (Additional file 1: Fig. S6A). The proportion mainly or exclusively smoking hand-rolled cigarettes was higher among less vs. more advantaged social grades (Fig. 4A). The absolute increase in use of hand-rolled cigarettes over time was larger among women of reproductive age from less advantaged social grades, rising by 25.6 percentage points (from 42.8% [37.5–48.3%] to 68.4% [61.5–74.5%]) compared with women of reproductive age from more advantaged social grades (+17.2 percentage points; from 35.3% [28.5–42.8%] to 52.5% [46.0–59.0%]; Fig. 4A). It was also considerably larger compared with men of the same age from less advantaged social grades (+13.3 percentage points; from 49.5% [44.2–54.8%] to 62.8% [55.9–69.2%]; Additional file 1: Fig. S6B) and with all adults from less advantaged social grades (+13.9 percentage points; from 44.2% [41.4–47.1%] to 58.1% [54.6–61.5%]; Fig. 4A).

There was a decline in current smokers’ mean level of dependence among women of reproductive age and a similar decline among all adults (Fig. 3B). These changes were concentrated among those from more advantaged social grades; among less advantaged social grades (who had consistently higher levels of dependence across the period) there was little change over time among women of reproductive age and an uncertain decline among all adults (Fig. 4B).

The rate of quit attempts among past-year smokers was slightly but consistently higher among women of reproductive age compared with the entire adult population. It decreased between 2013 and 2018, then increased between 2018 and 2023, such that there was little overall change from the start to the end of the study period among women of reproductive age or among all adults (Fig. 3C). There were divergent changes across social grades, with point estimates suggesting a potential decrease across the study period among those from more advantaged social grades and a potential increase among those from less advantaged social grades (in both women of reproductive age and all adults), although these changes were generally not statistically significant (Fig. 4C).

The success rate of quit attempts increased from 18.8% [14.1–24.5%] to 31.8% [25.6–38.7%] among women of reproductive age (Fig. 3D). A similar increase was observed among all adults over this period, from 16.8% [14.3–19.7%] to 25.4% [22.2–28.9%]. Increases in quit success may have been slightly greater among those from less vs. more advantaged social grades (e.g. among women of reproductive age: rising from 17.6% [11.9–25.3%] to 35.1% [25.8–45.8%] vs. 21.1% [14.1–30.4%] to 28.2% [21.0–36.7%], respectively), but this difference was uncertain (Fig. 4D).

## Discussion

Among women of reproductive age in England, there were notable changes in smoking, use of non-combustible nicotine products, and quitting activity between October 2013 and October 2023. Smoking prevalence decreased among those from less advantaged social grades but appeared to increase among those from more advantaged social grades. In contrast, among all adults, and among men of the same age, smoking prevalence remained relatively stable among those from more advantaged social grades. Changes in use of other nicotine products among women of reproductive age were more similar to those observed among other adults. The prevalence of vaping more than tripled, while use of NRT remained stable. Use of HTPs and pouches increased slightly in recent years but remained rare. However, there was a particularly pronounced increase among women of reproductive age in the proportion of smokers mainly or exclusively smoking hand-rolled cigarettes, with smaller increases observed among all adults and men of the same age. The mean level of dependence declined among more but not less advantaged social grades. The rate of quit attempts was consistently higher among women of reproductive age, but did not change substantially overall. The success rate of quit attempts increased by a similar amount among women of reproductive age and all adults.

Our data indicate there has been a rise in smoking prevalence among more advantaged women of reproductive age in England over the past decade. This is a different pattern to the one we observed in the general adult population and among men of the same age, where smoking prevalence declined overall and was relatively stable among the more advantaged social grades. This identifies more advantaged women of reproductive age as a group that may benefit from targeted intervention to prevent the uptake of (or relapse to) smoking. Trends among women from less advantaged social grades were more encouraging, showing a decline in smoking. As a result, inequalities in smoking among women of reproductive age have narrowed over this period. While reducing inequalities is an important public health priority, this would ideally be achieved by accelerating the decline in smoking prevalence among less advantaged groups, rather than stalling or reversing progress among those who are more advantaged.

Previous analyses of data from the Smoking Toolkit Study suggested that smoking rates may have increased among young adults in England during the early stages of the COVID-19 pandemic [21, 33]. However, the uncertain rise in smoking we observed among more advantaged women of reproductive age does not appear to have been driven by the pandemic: changes in smoking prevalence since the start of the pandemic were similar in this group to those among all adults and men of the same age from more advantaged social grades. Rather, the trends diverged prior to the pandemic. The reasons for this are unclear.

It is also unclear whether the uncertain rise in smoking prevalence among more advantaged women of reproductive age was driven by increased uptake among never smokers or relapse among former smokers (which is particularly common in the post-partum period [34]). We observed a decline in smokers’ mean level of dependence in this group of women. We speculated that there may have been an increase in non-daily smoking (e.g. social smoking) which is typically associated with lower levels of dependence [35]. Recent studies have documented increases in the prevalence of social smoking and non-daily smoking among adults in England [36, 37]. However, an exploratory analysis showed no notable differences in trends in the prevalence of non-daily smoking between women of reproductive age and all adults, or across social grades. Further research is needed to understand the reasons for this possible rise in smoking among more advantaged women.

Alongside the change in the mean level of dependence, we also observed a shift in the main type of cigarettes being smoked — away from manufactured cigarettes towards hand-rolled cigarettes. While this change in product choice was observed across all adults who smoked (as has been documented elsewhere [36]), it was more pronounced among women of reproductive age from 2019 onwards than among all adults or men of the same age — particularly among women from less advantaged social grades. It is possible that differences were driven by differing financial pressures associated with the COVID-19 pandemic and the ongoing cost-of-living crisis. The pandemic exacerbated gender inequalities, with women experiencing higher rates of job loss, taking on a disproportionate share of housework, childcare, and home-schooling responsibilities, and experiencing greater stress [38–44]. Job sectors in which women are overrepresented have done particularly badly since 2010, for example, with teaching and nursing pay freezes and creative sector cuts [45, 46]. In addition, the pandemic and the cost-of-living crisis worsened socioeconomic inequalities, hitting already disadvantaged groups harder [41, 47–49], which will have reduced their disposable income to spend on tobacco. These financial pressures probably contributed to the reduction in smoking prevalence among women from less advantaged social grades and encouraged those who did not stop to switch to hand-rolled products (which are considerably cheaper than manufactured cigarettes [50, 51]) as a way to afford to continue to smoke.

We also observed changes in quitting activity among women of reproductive age who smoked. There were increases in the rate of quit attempts and the success rate of quit attempts in recent years, with the increase in quit attempts reversing a declining trend in the early part of this study period. It is possible these changes were driven by an increased public health focus on reducing rates of smoking in pregnancy [7]. However, they largely mirrored changes observed among all adults, which were likely linked to the COVID-19 pandemic. A recent study found the start of the Covid-19 pandemic was associated with sustained increases in quitting among adults in England [33]. Our data show a similar pattern among women of reproductive age.

In addition to these changes in smoking and quitting activity, there were also changes in the use of non-combustible nicotine products. In particular, there was a substantial increase in vaping. In 2013, one in 20 women of reproductive age was a current vaper. By 2023, this number had risen to one in five. This finding is consistent with recent data showing a rapid rise in the uptake of vaping among young adults since a new generation of disposable vapes became popular from spring 2021 [52–54]. It appears to be an age- rather than gender-related phenomenon: although we saw a greater rise in vaping among women of reproductive age compared with the general adult population, an unplanned analysis showed the rise was similar to that observed among men of the same age. Use of NRT remained low (~3%) and stable over time and use of HTPs and nicotine pouches increased but remained rare (< 1%).

Strengths of this study included the large, nationally representative sample and repeated assessment of a range of smoking, nicotine use, and smoking cessation behaviours. There were also limitations. The rise in smoking prevalence among more advantaged women of reproductive age was uncertain, with a small overlap in the 95% confidence intervals for estimates of prevalence at the start (10.2–13.5%) and end (13.4–16.6%) of the study period. Further research is needed to confirm this finding. Women of reproductive age are not a homogenous group and the trends we have reported may differ according to age, lived circumstances, including relationship status (since living with a partner who smokes is a key predictor of postpartum relapse [55]), by preparedness or plans to have children, as well as the relative stresses experienced by sociodemographic subgroups. Further research could dig deeper into the trends we have observed, looking at differences between younger and older women of reproductive age, those with and without children, and those working across different sectors (e.g. those that have experienced substantial cuts or pay freezes in recent years). Qualitative research would be useful to provide insight into why smoking may have risen among women of more advantaged social grades and the extent to which transitioning to hand-rolled cigarettes plays a role in maintaining smoking. Another limitation was that quitting outcomes were self-reported and relied on recall of the past 12 months, but there is no reason to expect recall to differ across the time series. In addition, success of quit attempts was defined as continuous abstinence from quit date to the time of the survey, rather than abstinence over a defined period (e.g. 6 months). Finally, while the survey was representative of adults in households in England, it excluded people experiencing homelessness or living in institutions, who typically have much higher rates of smoking and living in situations in which women typically have worse health outcomes [56–58]. In addition, we used a hybrid sampling approach rather than random probability sampling — although comparisons with other sources suggest the survey recruits a nationally representative sample and produces similar estimates of key smoking variables [20, 59].

## Conclusions

While there has been a decline in smoking prevalence among women of reproductive age from less advantaged social grades over the past decade, smoking rates appear to have risen among women from more advantaged social grades. Across social grades, there have been substantial increases in the proportion of women of reproductive age who vape and shifts from use of manufactured to hand-rolled cigarettes among those who smoke. These changes have been more pronounced than those observed in the general adult population over the same period. Use of other non-combustible nicotine products among women of reproductive age remains low and does not differ substantially from the general adult population.

### Supplementary Information

**Additional file 1:Fig. S1.** Trends in the prevalence of smoking among women of reproductive age compared with men of the same age (18-45 years) in England, October 2013 to October 2023 – overall and stratified by occupational social grade. **Fig. S2.** Trends in the prevalence of non-daily smoking among women of reproductive age compared with all adults in England, October 2013 to October 2023 – overall and stratified by occupational social grade. **Fig. S3.** Trends in the prevalence of vaping among women of reproductive age compared with men of the same age (18-45 years) in England, October 2013 to October 2023 – overall and stratified by occupational social grade. **Fig. S4.** Trends in the prevalence of dual use of tobacco and non-combustible nicotine among women of reproductive age compared with all adults in England, October 2013 to October 2023 – overall and stratified by occupational social grade. **Fig. S5.** Trends in the prevalence of dual use of tobacco and non-combustible nicotine among women of reproductive age compared with men of the same age (18-45 years) in England, October 2013 to October 2023– overall and stratified by occupational social grade. **Fig. S6.** Trends in the proportion of current cigarette smokers mainly smoking hand-rolled (vs. manufactured) cigarettes among women of reproductive age compared with men of the same age (18-45 years) in England, October 2013 to October 2023 – overall and stratified by occupational social grade. **Table S1.** Smoking, use of non-combustible nicotine products, smoking characteristics, and quitting activity among women of reproductive age compared with all adults in England, 2013/14 to 2022/23. **Table S2.** Modelled estimates of changes in non-daily smoking and dual use of tobacco and non-combustible nicotine among women of reproductive age compared with all adults in England, from October 2013 to October 2023.

## Data Availability

Data are available from the corresponding author on reasonable request.

## Abbreviations

HTP: Heated tobacco product
NRT: Nicotine replacement therapy
RQ: Research question
