# Supplementary material for: Characterising smoking and nicotine use behaviours among women of reproductive age: a 10-year population study in England
Source: BMC Med. 2024 Apr 18;22:99. doi: 10.1186/s12916-024-03311-4 (PMC11025250; doi:10.1186/s12916-024-03311-4)
Supplement: Supplementary file 1 — Additional file 1:Fig. S1. Trends in the prevalence of smoking among women of reproductive age compared with men of the same age (18-45 years) in England, October 2013 to October 2023 – overall and stratified by occupational social grade. Fig. S2. Trends in the prevalence of non-daily smoking among women of reproductive age compared with all adults in England, October 2013 to October 2023 – overall and stratified by occupational social grade. Fig. S3. Trends in the prevalence of vaping among women of reproductive age compared with men of the same age (18-45 years) in England, October 2013 to October 2023 – overall and stratified by occupational social grade. Fig. S4. Trends in the prevalence of dual use of tobacco and non-combustible nicotine among women of reproductive age compared with all adults in England, October 2013 to October 2023 – overall and stratified by occupational social grade. Fig. S5. Trends in the prevalence of dual use of tobacco and non-combustible nicotine among women of reproductive age compared with men of the same age (18-45 years) in England, October 2013 to October 2023– overall and stratified by occupational social grade. Fig. S6. Trends in the proportion of current cigarette smokers mainly smoking hand-rolled (vs. manufactured) cigarettes among women of reproductive age compared with men of the same age (18-45 years) in England, October 2013 to October 2023 – overall and stratified by occupational social grade. Table S1. Smoking, use of non-combustible nicotine products, smoking characteristics, and quitting activity among women of reproductive age compared with all adults in England, 2013/14 to 2022/23. Table S2. Modelled estimates of changes in non-daily smoking and dual use of tobacco and non-combustible nicotine among women of reproductive age compared with all adults in England, from October 2013 to October 2023. [file 12916_2024_3311_MOESM1_ESM.docx]

**Table S1.** Smoking, use of non-combustible nicotine products, smoking characteristics, and quitting activity among women of reproductive age compared with all adults in England, 2013/14 to 2022/23

|  | **2013/14** | **2014/15** | **2015/16** | **2016/17** | **2017/18** | **2018/19** | **2019/20** | **2020/21** | **2021/22** | **2022/23** |
| --- | --- | --- | --- | --- | --- | --- | --- | --- | --- | --- |
|  |  |  |  |  |  |  |  |  |  |  |
| **Current smoking**, % [95%CI] |  |  |  |  |  |  |  |  |  |  |
| Women of reproductive age | 20.9 [19.6–22.1] | 21.9 [20.6–23.2] | 21.2 [20.0–22.4] | 20.3 [19.0–21.5] | 20.1 [18.9–21.3] | 17.8 [16.6–19.0] | 19.2 [17.9–20.6] | 20.0 [18.7–21.3] | 19.4 [18.1–20.7] | 18.2 [16.9–19.5] |
| All adults | 19.3 [18.7–19.9] | 19.4 [18.8–20.0] | 19.0 [18.4–19.5] | 17.7 [17.1–18.3] | 18.0 [17.5–18.6] | 16.2 [15.6–16.7] | 16.1 [15.5–16.7] | 16.6 [16.0–17.2] | 16.9 [16.3–17.5] | 16.7 [16.1–17.3] |
|  |  |  |  |  |  |  |  |  |  |  |
| **Current vaping**, % [95%CI] |  |  |  |  |  |  |  |  |  |  |
| Women of reproductive age | 5.8 [5.1–6.5] | 5.8 [5.0–6.5] | 6.1 [5.3–6.8] | 6.0 [5.3–6.8] | 6.2 [5.4–6.9] | 4.4 [3.8–5.1] | 6.9 [6.0–7.8] | 7.9 [7.0–8.8] | 12.5 [11.4–13.6] | 16.2 [15.0–17.5] |
| All adults | 5.0 [4.6–5.3] | 5.5 [5.1–5.8] | 5.8 [5.5–6.2] | 5.6 [5.2–5.9] | 5.7 [5.4–6.1] | 5.3 [4.9–5.6] | 5.9 [5.6–6.3] | 6.8 [6.4–7.2] | 8.4 [8.0–8.9] | 11.5 [11.0–12.0] |
|  |  |  |  |  |  |  |  |  |  |  |
| **Current NRT use**, % [95%CI] |  |  |  |  |  |  |  |  |  |  |
| Women of reproductive age | 3.0 [2.5–3.5] | 2.7 [2.2–3.2] | 2.2 [1.7–2.6] | 2.9 [2.4–3.4] | 2.3 [1.8–2.7] | 1.9 [1.5–2.3] | 2.4 [1.9–3.0] | 2.5 [2.0–3.0] | 2.2 [1.8–2.7] | 2.5 [2.0–3.0] |
| All adults | 3.2 [3.0–3.5] | 2.6 [2.3–2.8] | 2.5 [2.3–2.7] | 2.6 [2.4–2.8] | 2.5 [2.2–2.7] | 2.0 [1.8–2.2] | 2.3 [2.1–2.6] | 2.6 [2.4–2.9] | 2.9 [2.7–3.2] | 2.7 [2.5–3.0] |
|  |  |  |  |  |  |  |  |  |  |  |
| **Current HTP use**, % [95%CI] |  |  |  |  |  |  |  |  |  |  |
| Women of reproductive age | 0.0 [0.0–0.0] | 0.0 [0.0–0.0] | 0.0 [0.0–0.0] | 0.2 [0.1–0.3] | 0.1 [0.0–0.3] | 0.1 [0.0–0.2] | 0.1 [0.0–0.2] | 0.4 [0.2–0.6] | 0.5 [0.2–0.7] | 0.3 [0.1–0.5] |
| All adults | 0.0 [0.0–0.0] | 0.0 [0.0–0.0] | 0.0 [0.0–0.0] | 0.1 [0.0–0.1] | 0.1 [0.1–0.2] | 0.1 [0.0–0.1] | 0.1 [0.1–0.2] | 0.3 [0.2–0.3] | 0.3 [0.2–0.4] | 0.3 [0.2–0.3] |
|  |  |  |  |  |  |  |  |  |  |  |
| **Current pouch use**, % [95%CI] |  |  |  |  |  |  |  |  |  |  |
| Women of reproductive age | 0.0 [0.0–0.0] | 0.0 [0.0–0.0] | 0.0 [0.0–0.0] | 0.0 [0.0–0.0] | 0.0 [0.0–0.0] | 0.0 [0.0–0.0] | 0.0 [0.0–0.0] | 0.2 [0.0–0.3] | 0.3 [0.1–0.5] | 0.3 [0.1–0.5] |
| All adults | 0.0 [0.0–0.0] | 0.0 [0.0–0.0] | 0.0 [0.0–0.0] | 0.0 [0.0–0.0] | 0.0 [0.0–0.0] | 0.0 [0.0–0.0] | 0.0 [0.0–0.0] | 0.2 [0.2–0.3] | 0.4 [0.3–0.5] | 0.4 [0.3–0.5] |
|  |  |  |  |  |  |  |  |  |  |  |
| **Mainly smokes hand-rolled cigarettes**^1^, % [95%CI] |  |  |  |  |  |  |  |  |  |  |
| Women of reproductive age | 42.7 [39.4–46.1] | 43.6 [40.3–46.9] | 47.5 [44.2–50.9] | 47.2 [43.6–50.7] | 47.8 [44.3–51.3] | 51.3 [47.6–55.0] | 55.2 [51.0–59.4] | 53.5 [49.6–57.4] | 59.2 [55.1–63.3] | 60.4 [56.1–64.8] |
| All adults | 43.0 [41.3–44.8] | 44.8 [43.0–46.6] | 46.3 [44.5–48.1] | 47.3 [45.4–49.1] | 49.3 [47.5–51.1] | 48.4 [46.5–50.4] | 49.4 [47.2–51.5] | 50.4 [48.3–52.5] | 52.4 [50.2–54.6] | 53.3 [51.2–55.5] |
|  |  |  |  |  |  |  |  |  |  |  |
| **Level of dependence**^2^, geometric mean [95%CI] |  |  |  |  |  |  |  |  |  |  |
| Women of reproductive age | 1.19 [1.07–1.32] | 0.99 [0.87–1.11] | 0.93 [0.82–1.05] | 0.90 [0.78–1.02] | 0.79 [0.69–0.90] | 0.90 [0.78–1.03] | 0.90 [0.78–1.05] | 0.82 [0.71–0.95] | 0.75 [0.64–0.88] | 0.91 [0.78–1.05] |
| All adults | 1.11 [1.05–1.18] | 1.01 [0.95–1.08] | 0.99 [0.93–1.06] | 0.92 [0.86–0.99] | 0.88 [0.82–0.95] | 0.92 [0.86–0.99] | 0.84 [0.78–0.92] | 0.77 [0.71–0.83] | 0.79 [0.73–0.86] | 0.87 [0.81–0.94] |
|  |  |  |  |  |  |  |  |  |  |  |
| **Quit attempts**^3^, % [95%CI] |  |  |  |  |  |  |  |  |  |  |
| Women of reproductive age | 43.9 [40.7–47.1] | 37.5 [34.4–40.6] | 35.5 [32.4–38.5] | 37.2 [34.0–40.4] | 36.2 [33.0–39.4] | 28.7 [25.5–31.9] | 38.9 [35.2–42.5] | 39.3 [35.9–42.7] | 44.1 [40.6–47.7] | 43.1 [39.4–46.7] |
| All adults | 37.7 [36.1–39.3] | 32.7 [31.1–34.3] | 31.3 [29.7–32.9] | 33.2 [31.5–34.8] | 31.3 [29.7–33.0] | 28.8 [27.1–30.5] | 35.1 [33.2–37.0] | 36.7 [34.9–38.5] | 37.5 [35.7–39.3] | 37.0 [35.3–38.8] |
|  |  |  |  |  |  |  |  |  |  |  |
| **Quit success**^4^, % [95%CI] |  |  |  |  |  |  |  |  |  |  |
| Women of reproductive age | 19.3 [15.5–23.2] | 16.4 [12.4–20.3] | 18.1 [13.9–22.4] | 19.6 [15.2–24.0] | 14.2 [10.4–18.0] | 16.2 [11.2–21.2] | 20.7 [15.9–25.5] | 24.0 [19.3–28.7] | 28.2 [23.5–33.0] | 31.1 [25.8–36.3] |
| All adults | 18.5 [16.3–20.6] | 16.5 [14.2–18.8] | 20.5 [18.0–23.1] | 18.4 [16.0–20.8] | 16.2 [13.9–18.4] | 14.2 [11.9–16.6] | 20.3 [17.6–23.0] | 23.4 [20.8–26.0] | 24.1 [21.5–26.7] | 26.2 [23.5–28.8] |
|  |  |  |  |  |  |  |  |  |  |  |

CI, confidence interval. HTP, heated tobacco products. NRT, nicotine replacement therapy.

^1^ Among current cigarette smokers. ^2^ Among current smokers. ^3^ Among past-year smokers. ^4^ Among past-year smokers who made a past-year quit attempt.
Years run from October–September.


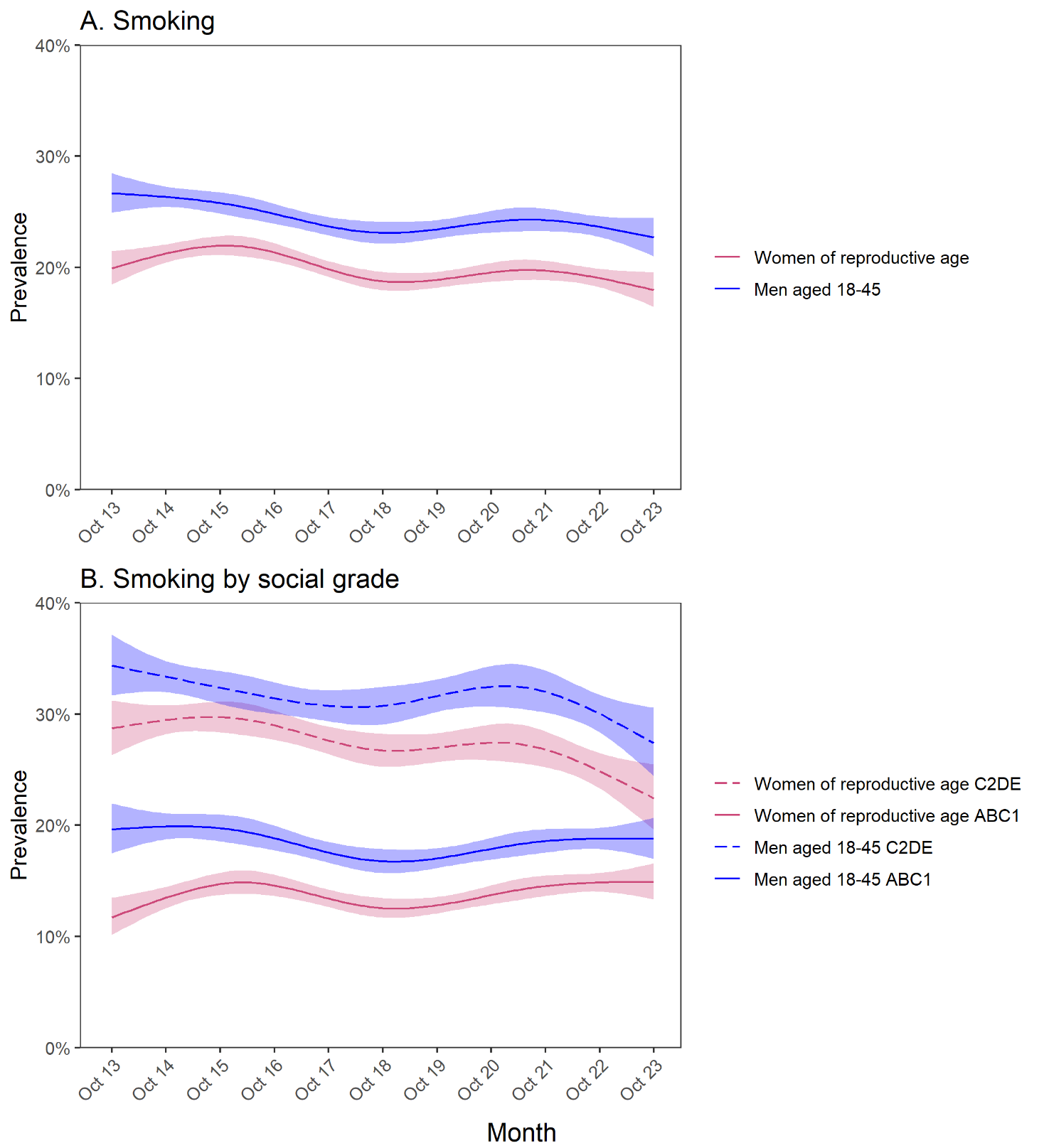


##### Figure S1. Trends in the prevalence of smoking among women of reproductive age compared with men of the same age (18-45 years) in England, October 2013 to October 2023 – overall and stratified by occupational social grade

ABC1 = more advantaged, C2DE = less advantaged. Lines represent modelled weighted prevalence by monthly survey wave, modelled non-linearly using restricted cubic splines (five knots). Shaded bands represent 95% confidence intervals.

##### **Table S2.** Modelled estimates of changes in non-daily smoking and dual use of tobacco and non-combustible nicotine among women of reproductive age compared with all adults in England, from October 2013 to October 2023

|  | **Overall** | | |  | **Social grades ABC1 (more advantaged)** | | |  | **Social grades C2DE (less advantaged)** | | |
| --- | --- | --- | --- | --- | --- | --- | --- | --- | --- | --- | --- |
|  | ***N*^1^** | **October 2013^2^** | **October 2023^2^** |  | ***N*^1^** | **October 2013^2^** | **October 2023^2^** |  | ***N*^1^** | **October 2013^2^** | **October 2023^2^** |
|  |  |  |  |  |  |  |  |  |  |  |  |
| **Non-daily smoking**, % [95%CI] |  |  |  |  |  |  |  |  |  |  |  |
| Women of reproductive age | 43,911 | 2.4 [1.9–3.0] | 4.4 [3.7–5.2] |  | 26,202 | 2.2 [1.6–3.1] | 4.4 [3.6–5.4] |  | 17,709 | 2.6 [1.8–3.6] | 4.3 [3.2–5.9] |
| All adults | 196,678 | 2.2 [1.9–2.5] | 3.5 [3.1–3.8] |  | 118,439 | 2.1 [1.8–2.5] | 3.4 [3.0–3.8] |  | 78,239 | 2.3 [1.9–2.8] | 3.6 [3.0–4.2] |
|  |  |  |  |  |  |  |  |  |  |  |  |
| **Dual use**, % [95%CI] |  |  |  |  |  |  |  |  |  |  |  |
| Women of reproductive age | 43,911 | 6.2 [5.3–7.2] | 9.5 [8.3–10.9] |  | 26,202 | 3.9 [3.0–5.2] | 7.6 [6.4–8.9] |  | 17,709 | 8.5 [7.1–10.2] | 12.3 [10.0–15.1] |
| All adults | 196,678 | 5.5 [5.0–5.9] | 7.2 [6.7–7.7] |  | 118,439 | 3.9 [3.4–4.4] | 5.7 [5.2–6.2] |  | 78,239 | 7.4 [6.7–8.2] | 9.1 [8.2–10.1] |
|  |  |  |  |  |  |  |  |  |  |  |  |

CI, confidence interval. HTP, heated tobacco products. NRT, nicotine replacement therapy.

^1^ Unweighted sample size for each analysis.

^2^ Data for October 2013 and October 2023 are weighted estimates of prevalence in these months (the first and last in the study period) from logistic regression with survey month modelled non-linearly using restricted cubic splines (five knots).

#####
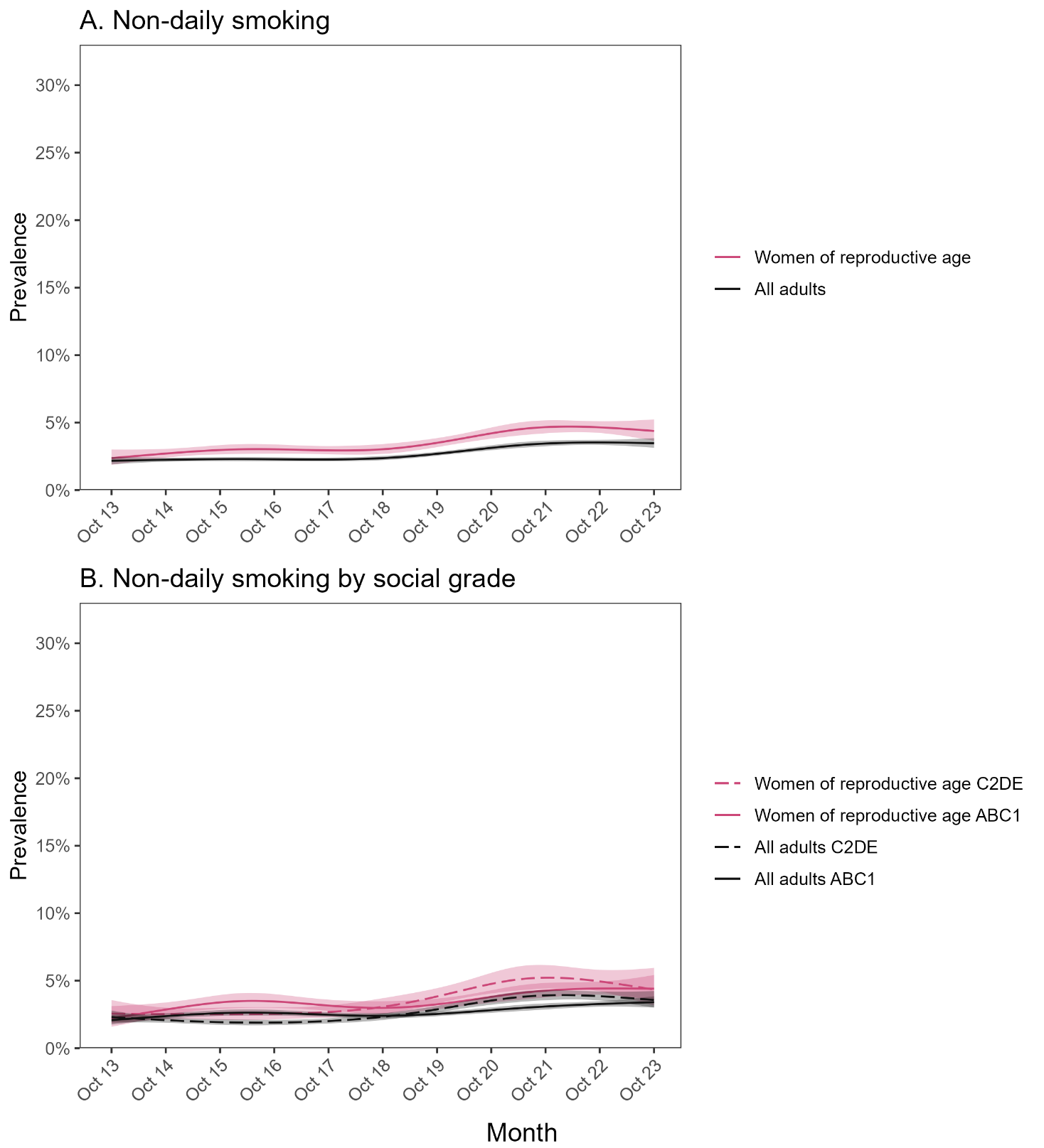
Figure S2. Trends in the prevalence of non-daily smoking among women of reproductive age compared with all adults in England, October 2013 to October 2023 – overall and stratified by occupational social grade

ABC1 = more advantaged, C2DE = less advantaged. Lines represent modelled weighted prevalence by monthly survey wave, modelled non-linearly using restricted cubic splines (five knots). Shaded bands represent 95% confidence intervals.


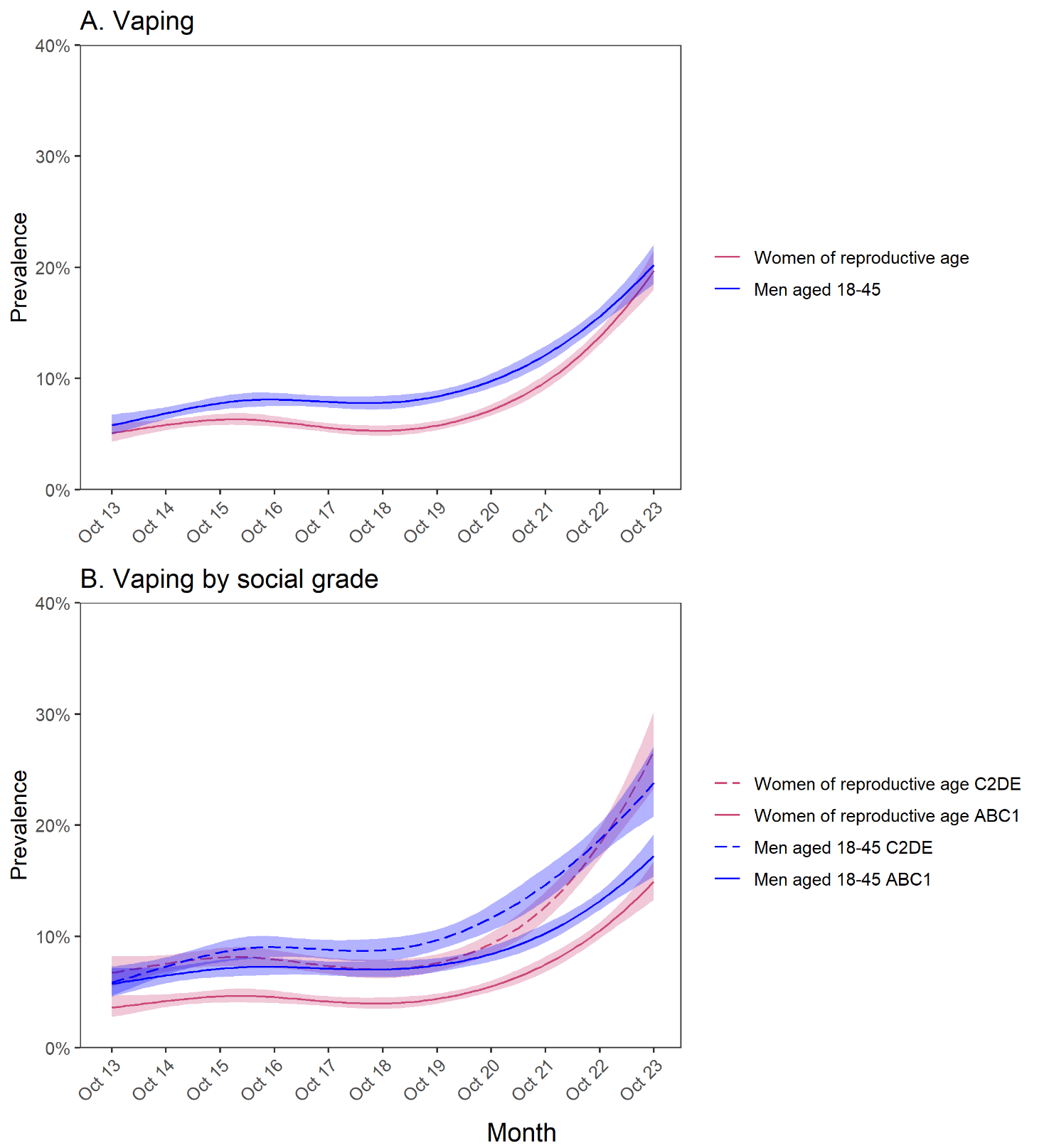


##### Figure S3. Trends in the prevalence of vaping among women of reproductive age compared with men of the same age (18-45 years) in England, October 2013 to October 2023 – overall and stratified by occupational social grade

ABC1 = more advantaged, C2DE = less advantaged. Lines represent modelled weighted prevalence by monthly survey wave, modelled non-linearly using restricted cubic splines (five knots). Shaded bands represent 95% confidence intervals.

#####
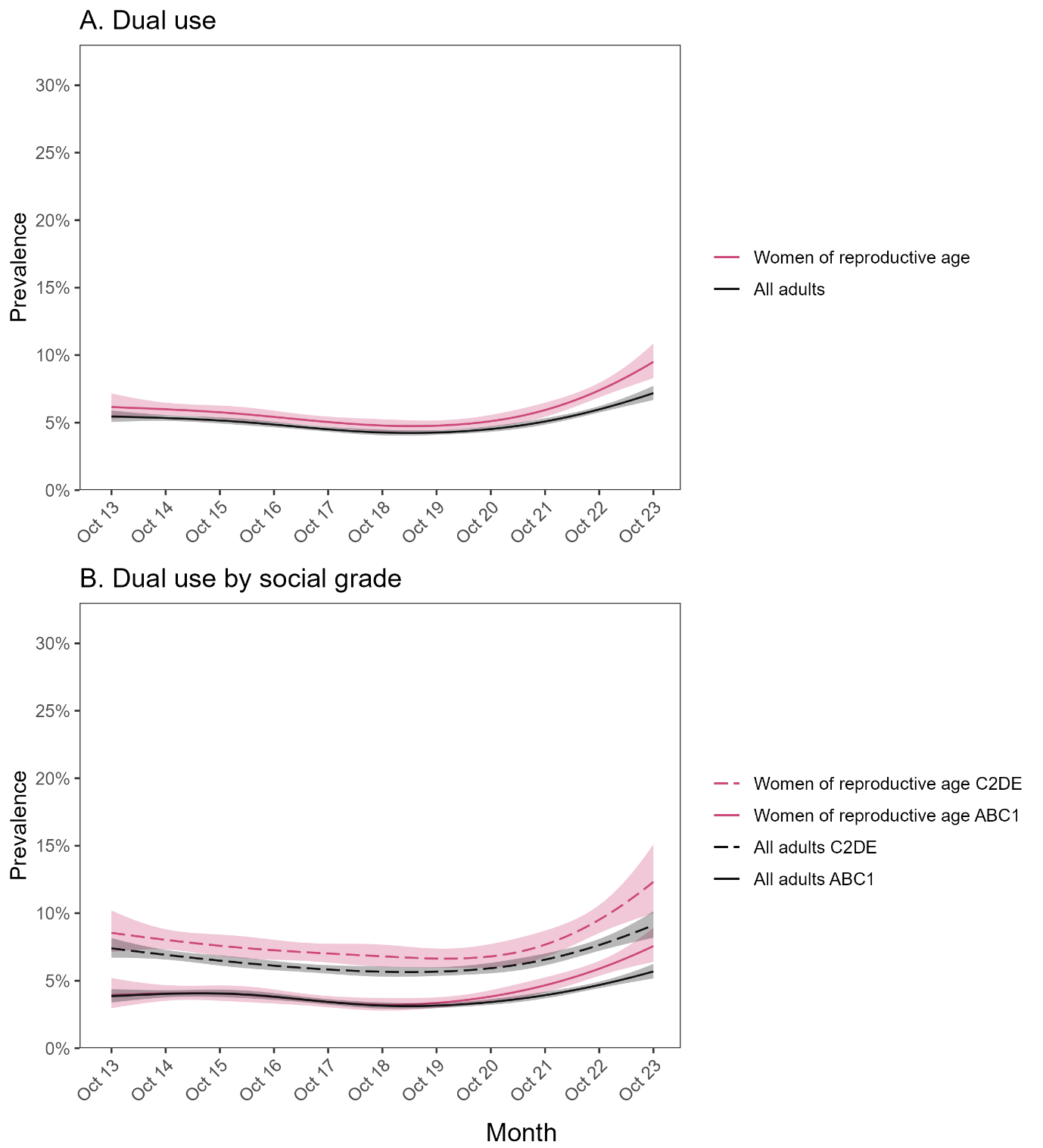


##### Figure S4. Trends in the prevalence of dual use of tobacco and non-combustible nicotine among women of reproductive age compared with all adults in England, October 2013 to October 2023 – overall and stratified by occupational social grade

ABC1 = more advantaged, C2DE = less advantaged. Lines represent modelled weighted prevalence by monthly survey wave, modelled non-linearly using restricted cubic splines (five knots). Shaded bands represent 95% confidence intervals.

#####
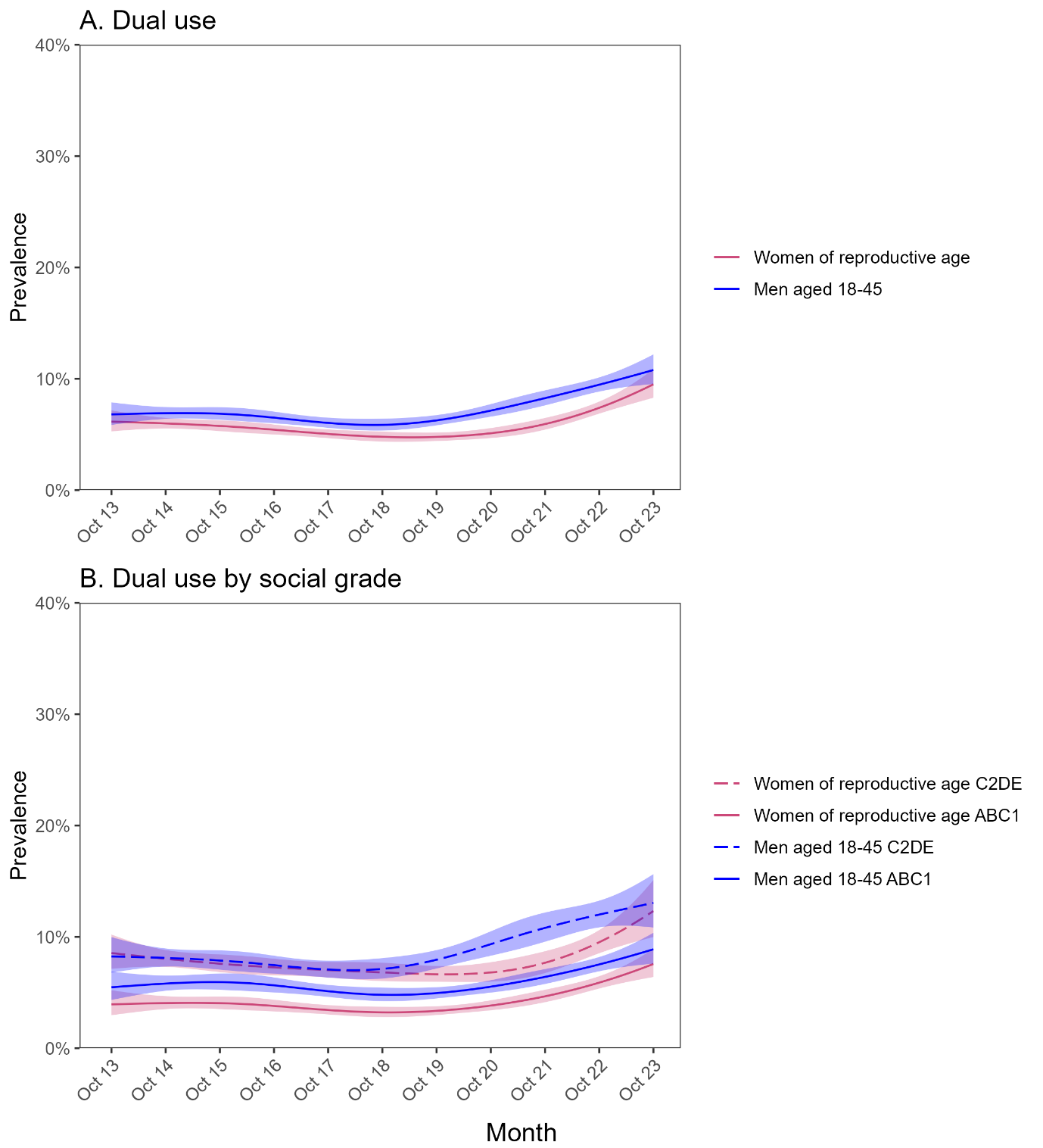


##### Figure S5. Trends in the prevalence of dual use of tobacco and non-combustible nicotine among women of reproductive age compared with men of the same age (18-45 years) in England, October 2013 to October 2023 – overall and stratified by occupational social grade

ABC1 = more advantaged, C2DE = less advantaged. Lines represent modelled weighted prevalence by monthly survey wave, modelled non-linearly using restricted cubic splines (five knots). Shaded bands represent 95% confidence intervals.


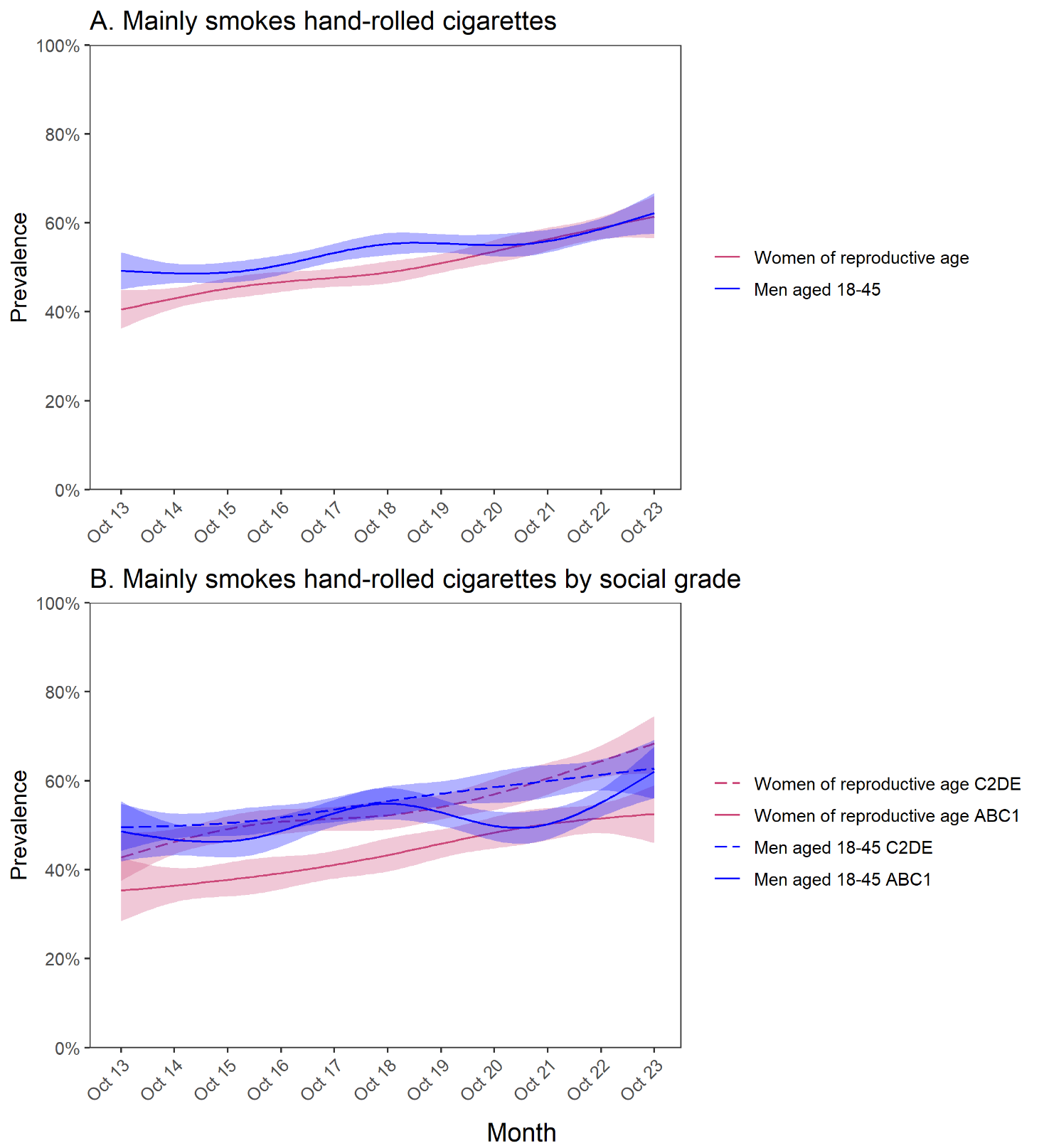


##### Figure S6. Trends in the proportion of current cigarette smokers mainly smoking hand-rolled (vs. manufactured) cigarettes among women of reproductive age compared with men of the same age (18-45 years) in England, October 2013 to October 2023 – overall and stratified by occupational social grade

ABC1 = more advantaged, C2DE = less advantaged. Lines represent modelled weighted prevalence by monthly survey wave, modelled non-linearly using restricted cubic splines (five knots). Shaded bands represent 95% confidence intervals.
